# Glenohumeral joint dislocation is rare in children with proximal humeral fractures: a descriptive study and literature review

**DOI:** 10.1186/s12891-021-04992-1

**Published:** 2022-01-05

**Authors:** Pan Hong, Saroj Rai, Ruikang Liu, Xin Tang, Jin Li

**Affiliations:** 1 Department of Orthopaedic Surgery, Union Hospital, Tongji Medical College, Huazhong University of Science and Technology, Wuhan, 430022 China; 2 Department of Orthopaedics and Trauma Surgery, Blue Cross Hospital, Tripureswor, Kathmandu, 44600 Nepal; 3 The First Clinical School, Tongji Medical College, Huazhong University of Science and Technology, Wuhan, China

**Keywords:** Humeral fracture, Children, External fixation, Glenohumeral joint dislocation

## Abstract

**Background:**

Glenohumeral dislocation combined with fracture of the proximal humerus is extremely rare in children, and this study aims to investigate its incidence in the pediatric population and review the treatment strategy for this condition.

**Methods:**

Between Jan 2014 and Jan 2019, 280 patients with unilateral proximal humeral fractures were retrospectively reviewed. Imaging and follow-up notes were reviewed for patients with a predilection for glenohumeral joint dislocation. Six (2.14%) patients between the ages of 5 and 10 years were confirmed as glenohumeral joint dislocation and included in the study. All these patients underwent closed reduction and external fixation under general anesthesia.

**Results:**

Out of 280 patients with proximal humeral fractures, only 6 patients, including 4 males and 2 females, were confirmed as glenohumeral joint dislocation. ROM was normal compared with the contralateral shoulder in every patient at the last follow-up. There was no case of radiological abnormality, including avascular necrosis or devascularization of the humeral head.

**Conclusions:**

Glenohumeral dislocation is a rare entity associated with the proximal humerus fracture in children, with an overall incidence in our case series was 2.14%.

Reduction and stabilization of such injury using an external fixator is a suitable choice for pediatric patients that failed closed reduction.

## Background

Fracture of the proximal humerus accounts for 2 to 5.4% of all fractures in the pediatric population [1, 2]. Nonsurgical treatment remains the primary choice for such fracture because of the tremendous remodeling potential in children [2, 3]. However, glenohumeral joint dislocation combined with fracture of the humerus is extremely rare in children. Open/closed reduction followed by surgical stabilization using Kirschner wire (KW) or elastic stable intramedullary nail (ESIN) has been reported in a handful of case reports [4]. Nelson G. et al. published a study of 220 pediatric proximal humerus fractures without any incidence of glenohumeral joint dislocation [5].

Shoulder fracture-dislocation in the adult population is another scenario. In the geriatric population, iatrogenic fracture during shoulder dislocation reduction is increasingly common [6]; however, this phenomenon is rarely observed in children. In adults with greater tuberosity fracture-dislocations, closed reduction attempts might be successful in some cases [7], but open reduction and internal fixation (ORIF) is usually warranted for displaced shoulder fracture-dislocation [8–10]. Reverse shoulder arthroplasty is generally indicated in the elderly with severely comminuted fracture-dislocations [11].

This study aims to investigate the prevalence of glenohumeral joint dislocation in children with fractures of the proximal humerus and review the treatment method we adopted to treat such injuries.

## Methods

Between January 2014 and January 2019, 280 patients with unilateral proximal humeral fractures were retrospectively reviewed (Table 1). The patients’ radiographic imaging and follow-up notes were reviewed in detail with a predilection for glenohumeral joint dislocation.

**Table 1:** Clinical characteristics of children with proximal humeral fractures

Parameter		Patients (n)	Patients (%) (*n* = 280)
Sex	Male	160	57.1
	Female	120	42.9
Side	Left	156	55.7
	Right	124	44.3
dislocation	with	6	2.1
	without	274	97.9
Mechanism	Falls	137	48.9
	Athletic Activity	106	37.9
	MVA	35	12.5
	Others	2	0.7
Classification	SH I	28	10
	SH II	32	11.4
	Metaphyseal	220	78.6
Neer-Horwitz Classification	I	190	67.9
	II	30	10.7
	III	22	7.9
	IV	38	13.5
Additional Imaging	With	41	14.6
	Without	239	85.4

Additional imaging examinations include CT, MRI or additional X-ray of different views other than AP and lateral view

*MVA* Motor vehicle accident, *SH* Salter Harris

Inclusion criteria were: Patients under the age of 14 years or less at the time of injury having a follow-up period of 3 weeks or more. Exclusion criteria were: (1) patients older than 14 years, (2) bilateral fractures, (3) open fractures or pathological fractures, including metabolic disorders, tumor, neuromuscular diseases, and (4) incomplete medical and radiological records.

Demographic information, including age, sex, mechanism of injury, and additional imaging, were collected from the hospital database. After the complete evaluation of the medical and radiological records of all 280 patients, 6 (2.14%) patients aged between 5 and 10 years were confirmed to have glenohumeral joint fracture-dislocation and had been treated with closed reduction and external fixation (EF) under general anaesthesia. Patients’ guardians were contacted via phone calls or social media (WeChat APP) and were requested to visit the hospital for final evaluation.

Follow-up data, including length discrepancy of the humerus (LDH), range of motion (ROM) of the shoulder joint [12], and any complications, were recorded at every follow-up visit and the last follow-up visit. LDH was measured radiologically and clinically. Radiological LDH was measured as the difference of humeral length (the highest point of the humeral head to the lowest point of trochlea) between two arms on standard AP view. Clinical LDH was measured as hands together with back against the wall and measuring the difference between the tip of middle fingers. After a 2-year follow-up, LDH was measured clinically only. Active ROM was recorded during every visit using a goniometer by the attending surgeon and compared with the contralateral shoulder. Less than 5 degrees difference of ROM than the contralateral shoulder was considered normal. The shoulder function was evaluated using American Shoulder and Elbow Surgeons subjective shoulder scale (ASES) [13].

### Surgical technique for glenohumeral joint dislocation with proximal humeral fractures in children

**Fig. 1:**
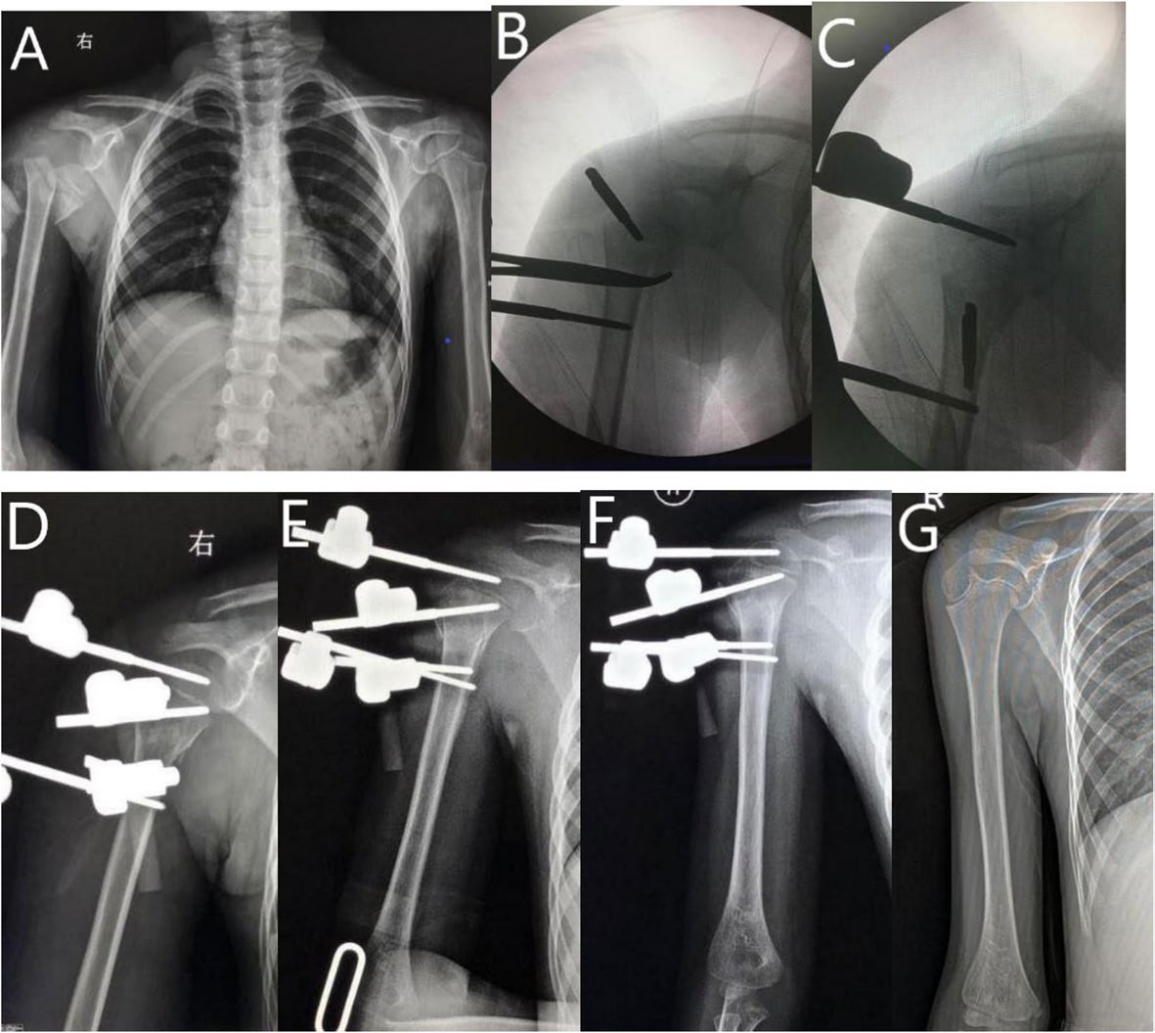
6-year-old girl with proximal humeral epiphyseal SH II fracture and shoulder dislocation. **A** Radiograph of humerus before surgery. **B** Radiograph of intraoperative reduction. **C** Radiograph of the shoulder joint after reduction. **D** Radiograph of the shoulder joint after surgery. **E** Radiograph of the humerus at 1-month follow-up. **F** Radiograph of the humerus at 2-month follow-up. **G** Radiograph of the humerus at 4-year follow-up

**Fig. 2:**
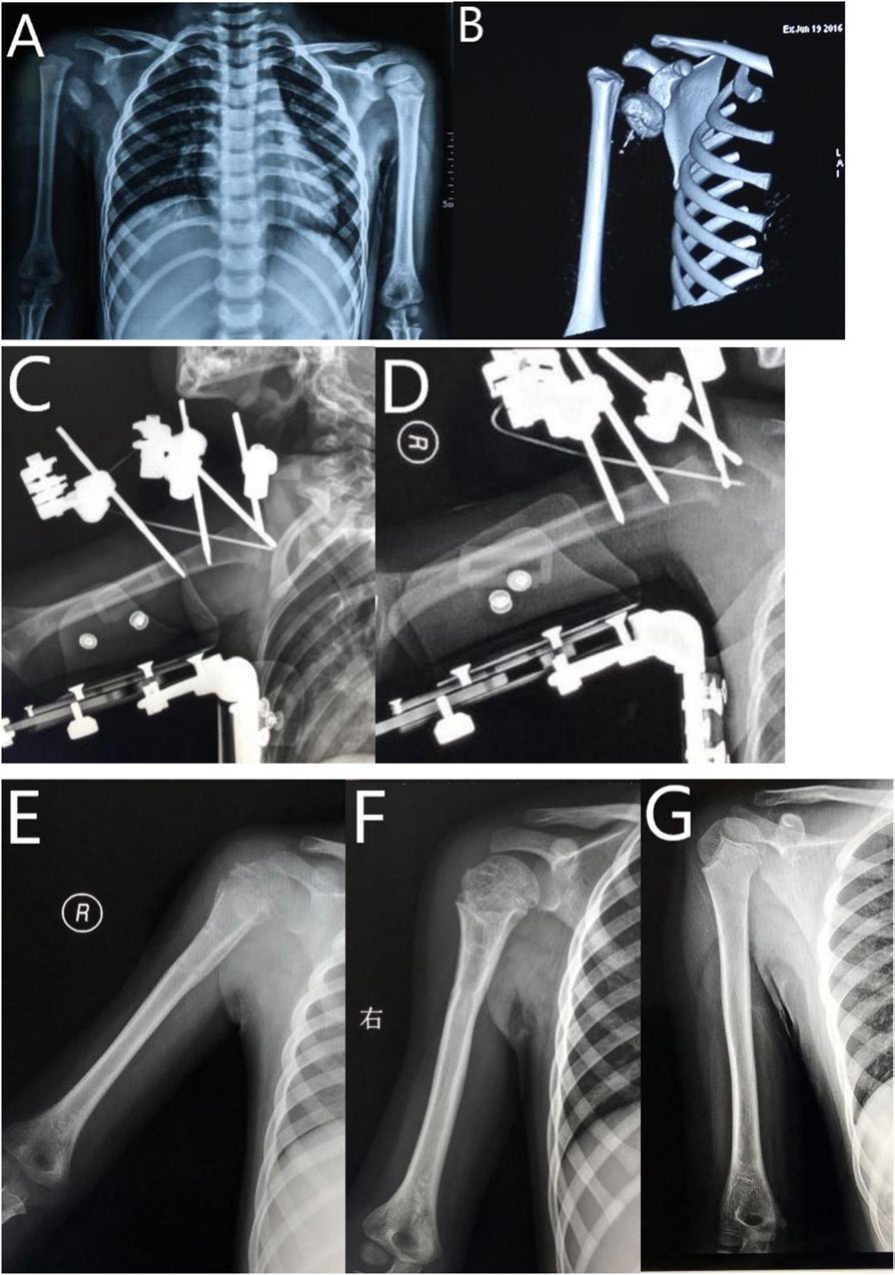
5-year-old boy with proximal humeral epiphyseal fracture SH I and shoulder dislocation. **A** Radiograph of humerus before surgery. **B** 3D reconstruction of CT scan. **C** Radiograph of the humerus after surgery. **D** Radiograph of the humerus at 1-month follow-up. **E** Radiograph of the humerus at 3-month follow-up. **F** Radiograph of the humerus at 6-month follow-up. **G** Radiograph of the humerus at 4-year follow-up

All the patients underwent surgery under general anesthesia in the supine position. The manual reduction was attempted in all cases before surgical stabilization. The procedure was performed under fluoroscopic guidance. Initially, the physis of the proximal humerus was identified. One or two Schanz pins were inserted into the metaphyseal region or epiphysis of the proximal fragment, and 2 Schanz pins were inserted into the humerus shaft. A small incision (5 mm) was made in the proximal humerus, followed by blunt dissection using hemostatic forceps until the bony surface was ascertained in order to avoid axillary nerve injuries. The Schanz pin was then introduced on the bony surface before drilling. The Schanz Pin was connected in the fixator, and then the glenohumeral dislocation could be easily reduced. Minor readjustment might be necessary to deliver a better fracture alignment (See Fig. 1). Occasionally, in patients with physeal injury, an additional KW is placed in the direction of humeral calcar across the physis to provide better stability (See Fig. 2). After the surgery, an arm sling or shoulder abduction brace was usually used for 3–4 weeks. Active ROM of the shoulder joint was initiated after removal of the sling or brace at 4 weeks. Subsequently, the patient was followed-up at every 2–3 months on an out-patient basis, and the external fixator was removed at 6–8 weeks postoperatively. Routinely, the active exercises were taught to the parents by the rehabilitation technician, and the patients were advised to perform home exercises under the guidance of their parents. Routine pin care was performed by the parents.

## Results

Among the available 6 patients, 4 were males and 2 were females (Table 2). All patients were classified as Neer-Horwitz IV. The average age of the patients was 7.8 (5–10) years. The etiology of fracture-dislocation of the proximal humerus in 4 patients was accidental falls, and that in 2 patients were motor vehicle accidents. The average duration from injury to surgery was 1.5 (1–2) days. Five patients had an anterior dislocation, while one suffered a posterior dislocation. Three patients had epiphyseal fractures, while remaining 3 patients had metaphyseal fractures. The average hospital stay was 3.3 (3–4) days (Table 2). The average follow-up period was 5 (3–7) years.

**Table 2:** Parameters of patients and operation

Patient	Age/Sex/Side	Mechanism	From injury to surgery, d	Dislocation	Fracture	Neer-Horwitz Classification	Reduction	Length of hospital stay, d	Follow-up
1	5/M/R	MVA	2	Anterior	Epiphyseal, SH I	IV	CR	3	4y
2	9/M/L	Fall	1	Anterior	metaphyseal	IV	CR	4	5y
3	6/F/R	MVA	1	Anterior	Epiphyseal, SH II	IV	CR	3	4y
4	10/F/L	Fall	2	Anterior	metaphyseal	IV	CR	4	3y
5	10/M/R	Fall	1	Posterior	Epiphyseal, SH II	IV	CR	3	7y
6	7/M/L	Fall	2	Anterior	Metaphyseal	IV	CR	3	7y

*M* male, *F* female, *R* right, *L* left, *MVA* motor vehicle accident, *CR* closed reduction

As shown in Table 3, ROM was normal in every patient at the last follow-up. Similarly, the average ASES was 96.8 (96–98) points at the last follow-up. The average LDH was 1.8 (1–3) mm at the last follow-up. The average angulation was 2.2 (1–3) degrees at the last follow-up without an apparent tendency of varus or valgus. Only one patient suffered from pin tract infection and was controlled successfully with oral antibiotics. There was no case of radiological abnormality, including avascular necrosis.

**Table 3:** Clinical parameters of follow-up

Patient	ROM	ASES	LDH, mm	Angulation, degree	PTI	Radiological abnormality
1	Normal	96	1	+ 2	Nil	Nil
2	Normal	97	2	+ 2	Nil	Nil
3	Normal	96	1	- 3	Nil	Nil
4	Normal	98	2	- 1	Yes	Nil
5	Normal	96	3	+ 3	Nil	Nil
6	Normal	98	2	+ 2	Nil	Nil

*ROM* range of motion, *ASES* American Shoulder and Elbow Surgeons, *LDH* length discrepancy of humerus, the length of contralateral humerus subtract the length of operative humerus, *PTI* pin tract infection

Normal ROM means less than 5 degrees loss of ROM compared with contralateral shoulder

Angulation = neck-shaft angle of contralateral limb subtract the neck-shaft angle of operative limb

## Discussion

AP and lateral radiograph of the shoulder joint are routine for the patient with a suspected proximal humeral fracture, and sometimes scapular-Y or axillary view might be required to rule out glenohumeral joint dislocation. However, because of the pain and noncompliance by the injured children, usually nonstandard AP and lateral view of the shoulder joint or proximal humerus was obtained during the out-patient visit. Fortunately, glenohumeral joint dislocation in children is rare [5]. Nevertheless, there were still 6 cases of shoulder dislocation in our study, and all cases were confirmed by additional CT scan. CT scan is generally not indicated in children with proximal humeral fractures since the incidence of shoulder dislocation is extremely rare. Most of the CT scans in our study were ordered by physicians in Emergency Department, not by an orthopedic specialist. All 6 patients with shoulder fracture-dislocation were classified as Neer-Horwitz IV, and therefore patients with severe fracture displacement require discretion and might warrant additional imaging. Moreover, for patients with high energy injuries such as motor vehicle accident, fall from height and serious contact sports injuries, advanced imaging is recommended to rule out the shoulder dislocation.

In our small series, the external fixator proved to be an effective method for treating traumatic fracture-dislocation of the shoulder in children after failed manual reduction, without the necessity of open reduction.

Shoulder dislocation is rare in children, and less than 2% of shoulder dislocation occurs in children younger than 10 years [14]. Although isolated fracture of the proximal humerus is not rare in children, so far, only 12 case reports of fracture-dislocation of the shoulder younger than 11-year-old have been reported in the literature [4, 15–25]. In the 12 case reports, 8 patients underwent open reduction (OR), and the remaining 4 patients underwent closed reduction followed by percutaneous pinning [16, 17, 19, 23]. Among 8 patients who underwent OR, KW was used in 6 cases [15, 18, 20–22, 24], ESIN was used in one patient [4], and no hardware was used in an 11-month-old child [25]. In our study, all 6 patients were reduced by closed method with the help of the external fixator.

It is challenging to reduce the fracture by the closed method in case of concomitant dislocation of the ipsilateral shoulder [5, 15, 19, 23]. When the fracture is reduced and stabilized, the dislocation can be easily reduced [5, 15, 23]. Therefore, we initially reduced the fracture and stabilized using an external fixator prior to the reduction of dislocation, as mentioned in a case report by Micic et al. [26]. However, the Schanz pin placement is more challenging in children with open growth plates. Utilization of both KW and ESIN have been reported in the treatment of proximal humeral fracture in children, but in fracture-dislocation injuries, KW and ESIN are not able to facilitate the reduction. In such a case, EF provides robust and accurate fixation, which is one of the reasons we utilized EF in fracture-dislocation.

Pin tract infection is a common complication in EF [27]. In our case series, 1 patient had a superficial infection that healed uneventfully with oral antibiotics and local wound care. There was no case of avascular necrosis (AVN) of the humeral head and growth disturbances. The reason might be that the placement of Schanz pin was performed under the guidance of fluoroscopy, and the reduction was performed in a closed fashion. In contrast, Wang et al. reported epiphyseal devascularization in a 10-year-old boy with fracture-dislocation of the shoulder [16], whereas Lee et al. reported AVN on the humeral head in a 16-year-old boy [28]. In both cases, open reduction was performed.

Bankart lesion has been reported in adolescents with shoulder dislocation [29]. However, no such lesion was observed in our study possibly because Bankart is more likely to happen in older children with shoulder dislocation in the absence of humeral fractures.

There were several limitations in our study. It was a retrospective study from a single medical center. Fracture-dislocation is a rare condition with 2.1% incidence in our study, and our treatment strategy using an external fixator is of the limited reference value because this technique requires a long learning curve and fluoroscopy guidance. Besides, ASES is not fully validated in children, and the clinical outcomes might not be thoroughly and meticulously measured [30].

## Conclusion

Glenohumeral dislocation is a rare entity associated with the proximal humerus fracture in children, with an overall incidence in our case series was 2.14%.

Reduction and stabilization of such injury using an external fixator is a suitable choice for pediatric patients that failed closed reduction.

## Data Availability

The datasets supporting the conclusion of this article are included within the article. Upon request, raw data can be provided by the corresponding author.

## Abbreviations

EF: External fixation
LDH: Length discrepancy of humerus
ROM: Range of motion
KW: Kirschner wire
ESIN: Elastic stable intramedullary nail
ASES: American Shoulder and Elbow Surgeons subjective shoulder scale
AVN: Avascular necrosis
OR: Open reduction
